# Hormonal and environmental signals guiding stomatal development

**DOI:** 10.1186/s12915-018-0488-5

**Published:** 2018-02-20

**Authors:** Xingyun Qi, Keiko U. Torii

**Affiliations:** Howard Hughes Medical Institute and Department of Biology, University of Washington, Seattle, WA 98195 USA

## Abstract

Stomata are pores on plant epidermis that facilitate gas exchange and water evaporation between plants and the environment. Given the central role of stomata in photosynthesis and water-use efficiency, two vital events for plant growth, stomatal development is tightly controlled by a diverse range of signals. A family of peptide hormones regulates stomatal patterning and differentiation. In addition, plant hormones as well as numerous environmental cues influence the decision of whether to make stomata or not in distinct and complex manners. In this review, we summarize recent findings that reveal the mechanism of these three groups of signals in controlling stomatal formation, and discuss how these signals are integrated into the core stomatal development pathway.

## Stomatal development—a brief overview

Stomata are micropores on the epidermis of above ground plant tissues, which serve as the passage for oxygen, carbon dioxide, and water between the external environment and internal plant tissues. Thus, stomata play a critical role for efficient photosynthesis, and in global carbon and water cycles [1, 2]. Upon opening, stomata facilitate the uptake of CO_2_ necessary for photosynthesis, but this process simultaneously enhances the evaporation of water through stomatal pores. To solve this dilemma, plants evolved sophisticated mechanisms to regulate stomata in coordination with various stimuli. In the short term, stomatal aperture is adjusted to optimize the balance between photosynthesis and transpiration [1]. In the long term, plants regulate stomatal development, responding to internal and external signals by changing the number of stomata [3]. Current understanding of stomatal movement upon diverse signaling can be sourced from a recent review [4]. Here, we will focus on the regulation of stomatal development.

**Fig. 1.**
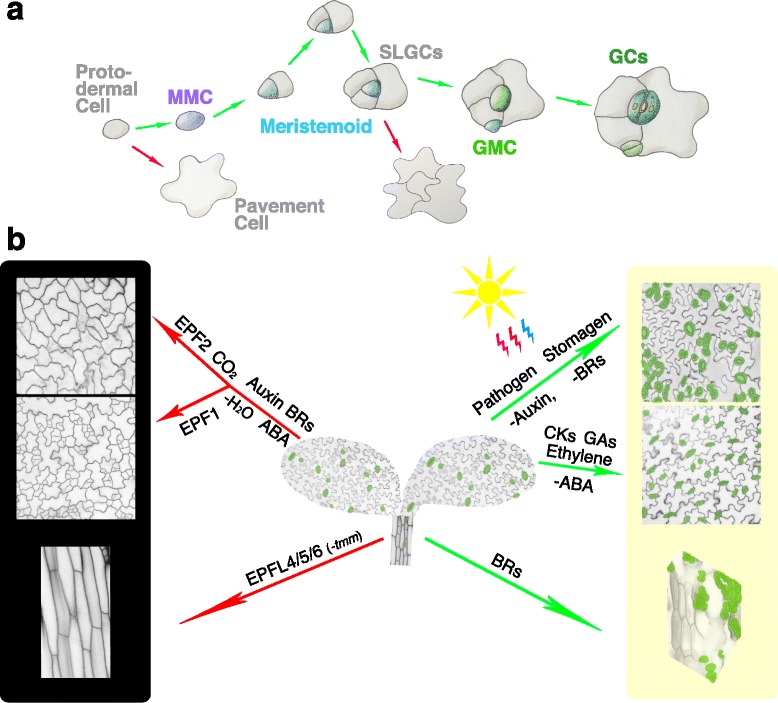
Summary of the effects that diverse range of signals have on stomatal development. **a** A cartoon showing stomatal cell-lineage transitions from a protodermal cell, a meristemoid mother cell (*MMC*), meristemoids undergoing asymmetric amplifying divisions and producing stomatal-lineage ground cells (*SLGCs*), and a guard mother cell (*GMC*) to a stoma with paired guard cells (*GCs*). A protodermal cell could differentiate into a pavement cell, and SLGCs could become pavement cells. Cartoons are modified from Han and Torii [11]. **b** An *Arabidopsis* seedling with stomata highlighted in *green* is in the *center*. Signals that negatively regulate stomatal development are shown on the *left*, indicated with *red arrows*. Signals that promote stomatal formation are shown on the *right*, indicated with *green arrows*. The *black* and *yellow boxes* indicate darkness (or signals that inhibit stomatal development) and light (or signals that promote stomatal development), respectively. When a signal is deficient, a minus sign is put in front of it. *Top left*: cotyledon epidermis with pavement cell only. *Middle left*: cotyledon epidermis with arrested meristemoids. *Bottom left*: hypocotyl epidermis with pavement cell only. *Top right*: cotyledon epidermis with clustered stomata. *Middle right*: cotyledon epidermis with high stomatal density. *Bottom right*: hypocotyl epidermis with clustered stomata. Confocal microscopy images of the cotyledon and hypocotyl epidermis of wild-type and various mutant seedlings were taken using a Leica SP5 WLL and false colored using Adobe Photoshop CS6

Studies on stomatal development have advanced greatly during the past decade. In the model plant *Arabidopsis*, stomata are produced through a stereotypical cell division and differentiation process, starting from a subset of protodermal cells called meristemoid mother cells (MMCs). MMCs initiate the stomata lineage by dividing asymmetrically to generate a small meristemoid and a large stomatal lineage ground cell (SLGC; Fig. 1a). The meristemoid, a precursor stem cell, can renew itself by one to three rounds of asymmetric division in an inward-spiral manner, producing a late meristemoid surrounded by SLGCs. The late meristemoid then differentiates into a guard mother cell (GMC), which will divide symmetrically once to generate a pair of guard cells surrounding a pore (Fig. 1a). The latest SLGC could also gain MMC cell fate and divide asymmetrically to generate a satellite stoma (Fig. 1a). The cell-state transitions above are controlled by the consecutive activities of several basic helix-loop-helix (bHLH) transcription factors, namely SPEECHLESS (SPCH), MUTE, and FAMA in coordination with their partner bHLH proteins SCREAM (SCRM, also known as ICE1) and SCRM2 [5–8]. SPCH is crucial for the entry asymmetric division of a meristemoid [6]. A careful analysis of the weak loss-of-function allele of *SPCH* also revealed its role of subsequently amplifying asymmetric divisions [7]. In contrast, the close relative of SPCH, MUTE, is required to terminate asymmetric division and promote differentiation, including symmetric division [7]. The last step of stomatal development is mediated by FAMA, which inhibits extra symmetric divisions in GMCs and promotes the GC identity [5]. Even though the developmental programs of stomatal formation differ among species, the bHLH transcription factors mentioned above represent the core module throughout land plants [9].

Stomatal patterning and density are two critical features for optimized stomatal function. During stomatal development, the one-cell spacing rule is tightly followed [3]. That means stomata are not formed in direct contact with each other, but with at least one non-stoma cell present between two stomata to enforce the proper opening and closure of the pore. Cell–cell communication is therefore essential in stomatal patterning [10]. Multiple signals, including secreted peptides that belong to the EPIDERMAL PATTERNING FACTORS (EPFs) family, plant hormones, and environmental stimuli, play important roles in concert with each other in both stomatal patterning and density (Fig. 1b) [3, 11, 12]. A well-known mitogen-activated protein kinase (MAPK) cascade consisting of YODA (YDA), MKK4/5/7/9, and MPK3/6 mediates these upstream signals by regulating the stability of the stomatal bHLH proteins in *Arabidopsis* [13, 14]. In this review, we will summarize recent findings on the signals that control stomatal development and discuss how their intricate signaling webs are integrated to bring about the differentiation of stomata in the model plant *Arabidopsis*.

## Stomatal development is controlled by secreted EPF peptide signals

So far, several EPF family members have been identified to play specific roles in distinct steps of stomatal development [15–22]. EPFs are plant peptide hormones that share a conserved structure with an N-terminal secretory signal peptide, followed by a predicted cleavage site and a mature peptide at the C-terminal end [19]. The predicted mature peptides contain six conserved cysteines that form intramolecular disulfide bonds, creating a loop region and a scaffold, and an additional two cysteines are found in some EPF family members [19]. Three cell surface leucine-rich repeat receptor kinases (LRR-RKs), ERECTA (ER), ER-LIKE 1 (ERL1), and ERL2, and one LRR receptor protein, TOO MANY MOUTHS (TMM), perceive the extracellular EPFs and transmit the signal into the cell [15–28]. A family of TMM suppressors called VAP-RELATED SUPPRESSOR OF TMM (VST) facilitate ER family signal transduction by forming complexes with integral endoplasmic reticulum membrane proteins [29].

The function of EPF2 has been intensively studied. *EPF2* is expressed in early precursors, MMCs and early meristemoids [16, 17]. The loss-of-function *epf2* mutant displays lots of small cells in the leaf epidermis, a phenotype also seen in plants overexpressing *SPCH* [16, 17]. Our genetic studies highlighted ER as a major receptor for EPF2 [26]. The kinase-deleted, dominant-negative form of ER phenocopied the *epf2* mutant, and furthermore conferred insensitivity to the EPF2 peptide application, together indicating that EPF2 and ER act in the same pathway with ER (the receptor) downstream of EPF2 (the ligand) [26]. Biochemical evidence together with the recently resolved crystal structure indicate that ER and TMM constitute a pre-formed receptor complex, which could bind EPF2, further supporting the above idea [26, 28]. Binding to the ER–TMM complex, EPF2 activates the downstream YDA MAPK cascade that eventually leads to the degradation of SPCH, the transcription factor that directly promotes the expression of EPF2 [10, 13, 14, 30, 31]. Consistent with this, exaggerated EPF2 signaling blocks entry to the stomatal lineage, resulting in an epidermis consisting of only pavement cells, a typical phenotype seen in the *spch* mutant [6, 16, 17].

EPF1 was the first member identified in the EPF family from a genome-scale screening on secreted peptides [15]. EPF1 shows specific expression in late meristemoids, GMCs, and young guard cells. The loss-of-function *epf1* mutation results in violation of asymmetric spacing division while EPF1 overexpression results in arrested meristemoids, phenocopying *mute* [15, 26]. It is thought that EPF1 is involved in meristemoid division polarity. Indeed, the polarized plasma membrane distribution of BREAKING OF ASYMMETRY IN THE STOMATAL LINEAGE (BASL), which predicts the position of the future division site, is defective in the *epf1* mutant [32]. It is possible that, in the absence of EPF1, paracrine signaling from the meristemoid to neighboring SLGCs becomes impaired, resulting in random orientation of secondary asymmetric spacing division. However, this cannot explain why application of EPF1 peptide confers arrested meristemoids (Fig. 1b) [15, 26]. EPF1 signal is primarily perceived by the ERL1–TMM receptor complex [27]. Furthermore, cell biological studies revealed that EPF1 is also involved in autocrine regulation in the late meristemoid and GMCs by targeting MUTE [27]. MUTE could promote *ERL1* expression, whereas ERL1 perceives EPF1 signal and inhibits MUTE activity. This negative feedback loop allows cells to elaborately regulate the amount of MUTE for proper stomatal differentiation [27].

EPF1 and EPF2 are two peptides with high similarity in sequence and structure, and they share their receptor complexes [26, 28]. Consistently, both EPF1 and EPF2 behave as negative regulators in stomatal development. However, excessive amounts of EPF2 lead to pavement cell-only epidermis, whereas the lines overexpressing *EPF1* show epidermis with no stomata but still asymmetric division divisions (Fig. 1b) [15–17, 26]. Loss-of-function mutants of *epf1* and *epf2* show distinct phenotypes [15–17]. Swapping promoter/coding regions of EPF1 and EPF2 fails to rescue the *epf1* or *epf2* mutant phenotype, respectively [16], indicating that EPF1 and EPF2 have distinct functions. An extracellular subtilisin-like serine protease, CO_2_ RESPONSE SECRETED PROTEASE (CRSP), which is essential for the generation of mature EPF2, could only cleave the pro-peptide of EPF2 but not EPF1, further confirming the specificity of EPF1 and EPF2 [33].

Stomagen/EPFL9 is expressed in mesophyll cells and promotes stomatal development (Fig. 1b) [18–20, 25, 28, 34]. Excessive Stomagen signal leads to an epidermis solely composed of stomata [18–20, 25, 34]. We demonstrated that Stomagen directly competes with EPF2 for binding to the ER receptor complex and inhibits the activation of downstream MAPK signaling [26]. Stomagen-mediated positive signaling can also be perceived by ERL1, implying Stomagen could compete with EPF1 as well for ERL1-containing complex [27]. The structural analysis of the ligand–receptor protein complexes supports the notion that Stomagen competes with EPF1 and EPF2 for binding to the same pocket created by ER family and TMM [28]. The antagonistic function between Stomagen and EPF2 is owing to their loop region rather than the scaffold [34]. Since the interaction between the ER family and TMM is constitutive, another receptor kinase is likely recruited upon EPF perception. Indeed, EPF peptide application triggers the association of ER family with SERK family co-receptors [35]. The loop region of EPFs may determine if such recruitment could happen or not.

The mutation in EPFL6/CHALLAH was identified as the suppressor of *tmm*, a mutant which displays stomata clusters on cotyledon and leaves, but does not form any stoma on hypocotyl (Fig. 1b) [21, 22, 36]. EPFL6 and its two close paralogs, EPFL4 and EPFL5, inhibit stomatal formation when they are ectopically overexpressed [22, 37], but the loss-of-function single mutants and even the triple mutant of the subfamily fail to show any stomatal phenotype, suggesting that they play a limited role in stomatal development under normal conditions [22]. In the *tmm* mutant background, however, knocking out EPFL4/5/6 altogether results in stomatal clusters in the hypocotyl [22]. These data suggest that TMM, instead of mediating the signal like EPF1/2, functions to reduce the signal of the EPFL6 subfamily. Unlike TMM, the ER family is still required to mediate EPFL4/5/6 signals [21, 22].

The crystal structure of the EPFs reveals that EPF1/2 and Stomagen could fit into the pocket made by the ER family and TMM, whereas EPFL4/5/6 show a high preference in binding to single ER family members, and this interaction is greatly compromised in the presence of TMM [28]. Sequence alignment indicates a conserved amino acid at the N-terminus of the mature peptide differs between the EPF1/2 and EPFL4/5/6 subfamilies [28]. The electrostatic potential of these amino acids may explain why the two subfamilies behave differently with respect to binding to TMM. It would then be interesting to test the ligand peptide activity after swapping these residues between EPF subfamilies. It should be noted that EPFL4/6 show expression in the stem endodermis (not in the epidermis), consistent with their higher-ordered mutant phenotype resembling the *er* mutant in terms of inflorescence architecture but with normal stomata in cotyledons and leaves [21, 22].

## Stomatal development is controlled by small chemical hormones

Plant hormones play vital roles in various aspects of plant development. Brassinosteroids (BRs) coordinate plant development and metabolism by promoting cell expansion and cell division [38–42]. Auxin influences plant growth by coordinating the placement and patterning of organs and cells, including root, shoot apical meristem, and floral primordia [43–45]. Abscisic acid (ABA) is a hormone produced in response to environmental factors, helping plants to adapt to stress conditions [46–48]. Stomata are essential for plant growth and adaption to the environment. With the mechanism of stomatal development being uncovered in the past decade, several recent studies have opened the door to understanding plant hormones’ functions in stomatal development (Fig. 1b).

### Brassinosteroids

BRASSINOSTEROID INSENSITIVE 1 (BRI1), a membrane-bound LRR-RK, is the receptor of BRs. When binding to BRs, BRI1 will recruit its co-receptor BRIASSOCIATED RECEPTOR KINASE (BAK1)/SERK3 and inactivate the GSK3/SHAGGY-like kinase BRASSINOSTEROID INSENSITIVE 2 (BIN2). BIN2 negatively regulates a set of downstream transcription factors, including BRASSINAZOLE RESISTANT 1 (BZR1), to prevent the BR-mediated gene expression [49, 50].

**Fig. 2.**
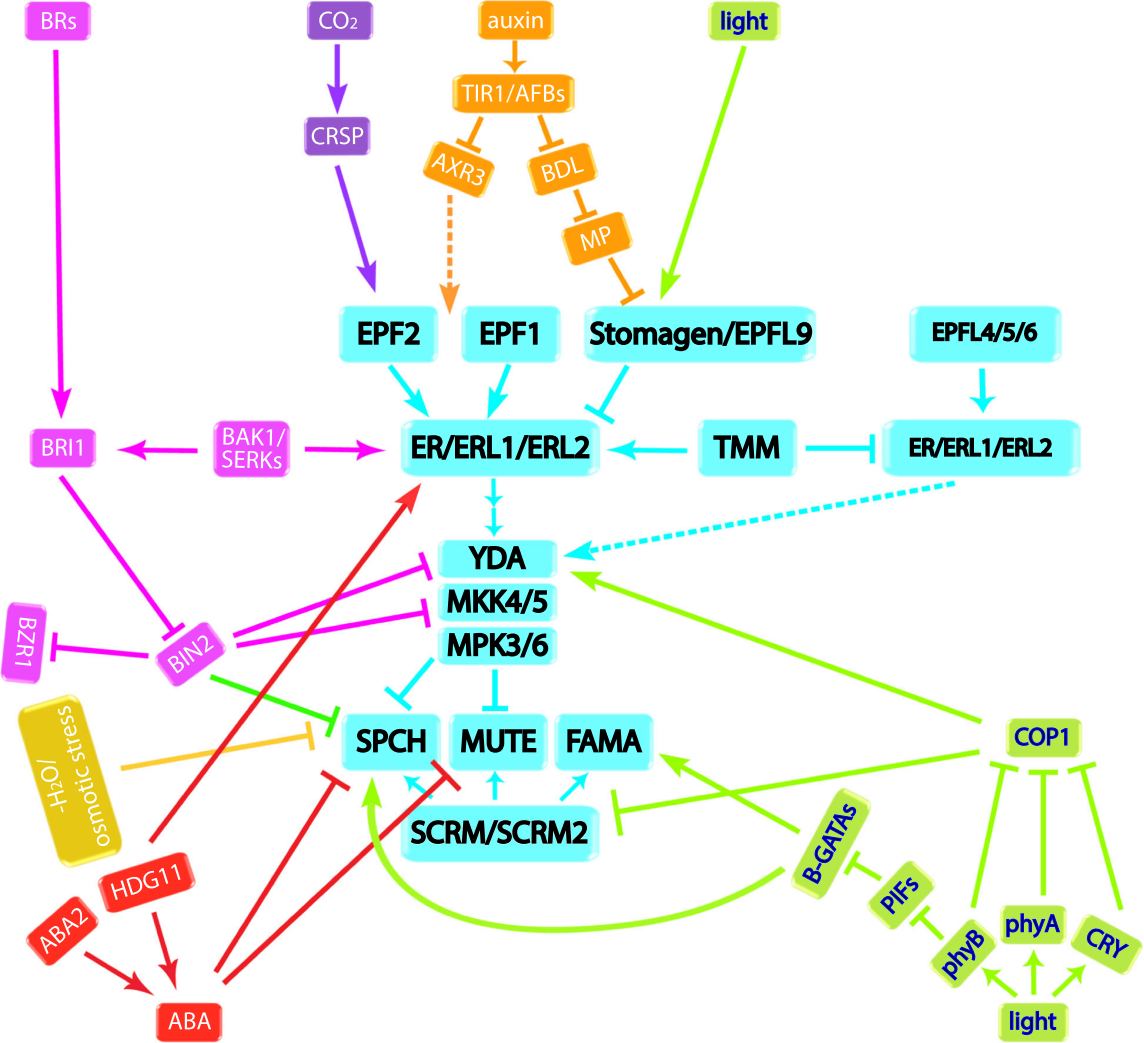
How hormonal and environmental cues are integrated into the core stomatal developmental pathway. Components involved in the same pathway are grouped with the same color. The experimentally confirmed steps are shown as *solid lines*, and the steps that are uncertain are shown as *broken lines*. An *arrow* indicates a positive regulation, while a ‘*T*’ indicates negative regulation

Several studies claim that BRs repress stomatal formation by interacting with the YDA MAPK cascade of the stomatal development pathway (Fig. 2) [51, 52]. Focusing on *Arabidopsis* cotyledons, it was discovered that *bri1* and dominant *bin2* exhibit stomatal clusters, whereas application of the most active BR hormone brassinolide (BL) reduces stomatal density [51]. A similar stomatal patterning defect was also observed in the first leaf pair [52], indicating a negative regulation of BRs on stomatal development. However, mutations in the downstream transcription factor BZR1 do not violate stomatal patterning [51]. In the core stomatal pathway, BIN2 acts in parallel with the ER-TMM receptors, but still requires the YDA MAPK cascade and the downstream stomatal transcription factors [51], implying that the MAPK cascade may be the target of BIN2 in the stomatal pathway. Indeed, BIN2 could inhibit YDA activity by phosphorylating its N-terminal regulatory domain [51]. In addition, BIN2 can specifically phosphorylate MKK4 at Ser-230 and Thr-234, which inhibits the downstream activation of MPK6 [52]. These studies suggest that BIN2 regulation on the YDA MAPK cascade could be the integration site of the two signaling pathways (Fig. 2).

In contrast, there are also studies supporting the idea that BR signaling promotes stomatal development (Fig. 2) [53, 54]. In the hypocotyl of the *bri1* mutant, or in plants in which BR biosynthesis is inhibited by brassinazole (BRZ), the number of stomata is greatly reduced; whereas when BR signaling is enhanced by applying BL or by overexpressing *BRI1*, the number of stomata is significantly increased in the hypocotyls [53]. Again, BIN2 is the integration point. The target of BIN2 in promoting stomatal development in the hypocotyls is SPCH. It has been shown that BIN2 can reduce the stability of SPCH protein by phosphorylation [53]. In support of this, the reduced stomatal numbers in both cotyledons and hypocotyls of *spch-5*, which has a missense mutation within the DNA binding domain of *SPCH*, could be partially suppressed by BL treatment [54]. Application of BL increases the amount of SPCH-5 protein and restores the expression of a set of SPCH target genes, including BASL and EPF2 [54].

The discrepancy between the above studies could be due to tissue specificity. Cotyledons and hypocotyls show distinct phenotypes when TMM is mutated [36]. It is implied that the ligand–receptor pairs in stomatal development differ in the two tissues, which possibly affects the downstream pathway, including the YDA MAPK cascade, in distinct manners [21, 22, 28]. The SERK family members, including BAK1/SERK3, bind to the ER family and TMM in an EPF-dependent manner, and higher-ordered *serk* mutants show stomatal clusters [35]. How plants balance the perception of BRs and EPFs at the receptor level in the cotyledon and hypocotyl is so far unclear. Notably, BR could repress chloroplast development and inhibit photomorphogenesis and photosynthetic gene expression [55–57]. Stomatal development should coordinate with these processes to optimize photosynthesis, which primarily happens in leaves. Indeed, it has been reported that stomatal development in the epidermal layer does couple with the cell development in the underlying mesophyll tissues to match leaf photosynthetic potential with gas exchange capacity [58]. It is possible that plants integrate additional tissue-specific signaling pathways when regulating the crosstalk between BR signaling and stomatal development. Sterols other than BRs also play roles in stomatal development, as revealed by a study on the sterol C-14 reductase gene FACKEL [59]. Since the underlying mechanism is still unknown, it is not clear if this signal has any influence on BR signaling in stomatal formation.

### Auxin

Auxin is a plant hormone that widely regulates plant development, but its role in stomatal development was reported only recently [60–63]. An interesting time-lapse experiment reveals that auxin activity changes over stomatal development [58]. Auxin activity is high in early stages but depleted from GMCs, probably via its efflux transporter PIN FORMED3 (PIN3), based on the strong expression of PIN3 in late meristemoids. In *pin* higher-order mutants or when PIN3 trafficking is interfered with, stomata form clusters, suggesting on-time exporting of auxin from meristemoids is critical for stomatal patterning [60].

The auxin signal in early steps of stomatal development may contribute to the amplifying asymmetric division step. The intracellular auxin could be perceived by the nuclear receptor TRANSPORT INHIBITOR RESPONSE 1 (TIR1)/AUXIN-BINDING F-BOX (AFB), which then binds to AUXIN/INDOLEACETIC ACID (Aux/IAA) proteins. Subsequent degradation of Aux/IAA releases the transcription factors AUXIN RESPONSE FACTORs (ARFs), resulting in auxin response [64]. Excess auxin from either exogenous application or genetic manipulation reduces the number of stomata and meristemoids, whereas auxin-deficient, auxin transport-deficient, or auxin signaling-deficient mutants exhibit stomatal clusters, suggesting a negative role of auxin in stomatal development [61–63]. Consistent with this, stabilizing the suppressor Aux/IAA BODENLOS (BDL/IAA12) or AUXIN RESISTANT3 (AXR3/IAA17) increases stomatal density, a similar phenotype to that produced by mutating MONOPTEROS (MP)/ARF5, the IAA-ARF pair of BDL [43–45, 61, 62]. The inhibition effect of this MP-involved auxin pathway on stomatal formation could be explained by its regulation of *STOMAGEN*. AuxRE elements, which MP shows strong binding activity to, are found in *STOMAGEN* promoter. MP inhibits the stomatal pathway by suppressing the expression of *STOMAGEN* in mesophyll cells (Fig. 2) [62].

The target of AXR3 remains unknown, but as it acts upstream of the ER family it is possible that AXR3 controls stomatal development by regulating the expression of other EPFs via an unknown ARF. It should be noted that the inhibition effect of auxin signaling on stomatal development by suppressing AXR3 only occurs when the light signal is absent [61]. Maybe light stabilizes AXR3, but probably not through the CONSTITUTIVELY PHOTOMORPHOGENIC1 (COP1)-mediated pathway, as it has been shown that the two proteins act independently in controlling the stomatal pathway [61]. Unlike the BDL-MP pathway, stomatal clusters are not formed in darkness when AXR3-mediated auxin signaling is deficient [61]. One explanation for this would be that MP-mediated auxin signaling plays a role in both stomatal density and stomatal patterning, probably by transcriptional regulation of downstream genes. Alternatively, it is possible that light signaling is also contributing to the phenotype in the BDL-MP study. In line with this, *STOMAGEN* expression could be stimulated by light [65]. It will be interesting to test if BDL or MP is regulated by light signaling and if their influence on stomatal development is related to light signaling.

### Abscisic acid

The role of ABA in stomatal physiology is well-known [66]. In addition to stomatal movement, ABA also affects stomatal development (Fig. 1b). Application of ABA reduces the number of stomata per leaf in wheat [67]. On the other hand, ABA promoted stomatal formation on the water-submerged leaves in *Potamogeton perfoliatus* [68], implying the complex ABA effect could be species dependent. In *Arabidopsis*, mutants defective in ABA metabolism or in ABA signaling display high stomatal density [69, 70], whereas defects in ABA catabolic enzymes result in fewer stomata [69], suggesting that ABA represses stomatal development. Indeed, the ABA biosynthesis mutant *aba2* displays prolonged expression of *SPCH* and *MUTE*. Double mutant analysis of *aba2* with *spch* and *mute* further revealed that ABA restricts stomatal-lineage divisions at the point of *SPCH*, upstream of *MUTE* [69]. In addition, HOMEODOMAIN GLABROUS11 (HDG11), which promotes ABA production, also activates the *ER* gene (Fig. 2) [71, 72], implying that ABA affects stomatal development at multiple levels.

### Other hormones (gibberellins, ethylene, cytokinins, and jasmonic acid)

Several other hormones are also reported to influence stomatal development (Fig. 1b). For example, gibberellin (GA) treatment of *Arabidopsis* plants causes stomatal density to increase in the hypocotyls [63]. *Arabidopsis* plants grown in medium supplemented with the exogenous ethylene precursor 1-aminocyclopropane-1-carboxylic acid (ACC) display increased stomatal density, whereas interrupting the ethylene-signaling pathway leads to reduced stomatal density, suggesting a positive role of ethylene in stomatal development [73]. The underlying molecular mechanism, however, remains elusive.

In tomato, enhancing cytokinin (CK) signaling increased stomatal density, but stomatal index and patterning remain unchanged. Further study revealed that the fundamental role of CK in this case is to promote epidermal cell division, rather than directly promoting stomatal development [74]. A stomatal-lineage transcriptome analysis in *Arabidopsis* revealed that a CK signaling component, *ARABIDOPSIS RESPONSE REGULATOR 16* (*ARR16*), and CK catabolic enzyme gene *CYTOKININ OXIDASE4* (*CKX4*) are highly and specifically enriched in the meristemoids [75], suggesting a possible role of CK in stomatal development. The same study identified a key transcription factor of jasmonic acid (JA) signaling, *JASMONATE-ZIM-DOMAIN PROTEIN 10* (*JAZ10*), highly and specifically expressed in the meristemoids [75]. Further studies are required to clarify if these signaling pathways directly contribute to stomatal development, and if so, how these signals integrate with the stomatal development pathway.

## Stomatal development is controlled by environmental factors

As stomata are the windows through which plants exchange gas and water with the environment, it is not surprising that environmental factors, especially light, CO_2_, and water, would influence stomatal development as feedback (Fig. 1b). An interesting mechanism revealed by a clever leaf-cuvette experiment is that light and CO_2_ levels perceived by mature leaves, which have more access to these environmental signals but less plasticity in stomatal development, could affect the stomatal density in young leaves [76]. A long-distance signal transmitted from mature leaves is predicted to control stomatal formation in expanding leaves. The photoreceptor Phytochrom B (phyB) could be one component in this process, as inducing phyB only in mature leaves resulted in an increase in stomatal index for non-induced young leaves under high light, whereas *phyB* mutant lost this systemic regulation on stomatal development [77]. Other than the systemic control, an increasing number of studies demonstrate that external signals could influence stomatal formation by integrating into the intrinsic stomatal developmental pathway at various steps [33, 65, 77–83].

### Light

Elevated light intensity promotes stomatal formation [65, 77, 80–83]. *Arabidopsis* perceives light signals using photoreceptors, including the cryptochrome (CRY) blue/UV-A light photoreceptors and the phytochrome (phy) red/far-red light photoreceptors [84–88]. Plants with mutations in these photoreceptors become insensitive to the corresponding light spectra and show reduced stomatal density [80–82]. Among the five phytochromes, PhyB plays the primary role in perceiving red light, and PhyA might be the sole photoreceptor perceiving far-red light to induce stomatal development [81, 82]. Although PhyB, PhyA, and the CRYs act additively in promoting stomatal formation, they all negatively regulate COP1, a repressor of light signaling that also inhibits stomatal development [82]. A null allele of *cop1* shows severe stomatal clusters resembling *yda* mutant. Introducing constitutively active delta N-YDA completely reverses the *cop1* stomatal cluster phenotype, exhibiting pavement cell only epidermis [82], suggesting YDA acts downstream of COP1. TMM, on the other hand, acts in parallel with COP1, as their double mutant shows an additive stomatal phenotype [82]. Like the case of BR signaling, YDA may be the integration point between light signaling and stomatal development pathways (Fig. 2). Further biochemical evidence of an interaction or regulator relationships between COP1 and YDA could help clarify the hypothesis.

Interestingly, it seems that YDA may not be the only integration point. A recent report shows that the E3 ubiquitin ligase COP1 also directly interacts with SCRM and SCRM2 in the dark, and this causes the degradation of SCRM proteins through ubiquitin/proteasome pathways [89]. Thus, in darkness, when COP1 is stable and active, SCRM proteins are degraded, thereby preventing stomatal differentiation (Fig. 2). Interestingly, SCRM accumulation is still light-responsive in the *yda-10* mutant [89]. Therefore, COP1-mediated degradation of SCRM proteins may not occur through the YDA pathway.

Another piece of evidence points to transcription factors at the integration point between stomatal development and light signaling. Light signal induces GATA factors of the B-subfamily (B-GATA) transcription factors to facilitate the expression of *SPCH* [83]. B-GATAs promote stomatal development in hypocotyls in a light-dependent manner. A quadruple B-GATA mutant, *gata-q*, hardly forms stomata in the hypocotyl epidermis regardless of light, indicating they are essential for the stomatal formation. Genetic analysis puts *B-GATAs* downstream of *EPFL4/5/6* and *TMM*, but upstream of *SPCH*, *MUTE*, and *FAMA* in the core stomatal pathway. In light signaling, B-GATAs are downstream of and suppressed by phytochrome-interacting factors (PIFs), the bHLH transcription factors that act to negatively regulate photomorphogensis [83, 90–93]. As transcription factors, B-GATAs promote *SPCH* expression by directly binding to its promoter (Fig. 2) [83]. The exaggerated stomatal differentiation caused by gain-of-function *scrm-D* mutant can be suppressed by the higher-order *gata-q* mutants [84]. Since *SCRM* is a direct SPCH target [31, 94], this suppression may be directly due to the reduced *SPCH* expression levels in *gata-q* hypocotyls. It has been reported that the expression of *STOMAGEN* is also induced by light [65], and B-GATAs are expressed in mesophyll as well [83]. As such, it will be interesting to test if B-GATAs also regulate *STOMAGEN* expression during stomatal development. Notably, the *pif-q* mutant develops more stomata than wild type in darkness, and two B-GATAs are upregulated in *pif-q* [83]. Where to place the PIFs in the intrinsic stomatal development pathway remains an open question.

### Carbon dioxide

CO_2_ is a substrate for photosynthesis that is absorbed through stomata. A survey of 100 species revealed that elevated concentrations of CO_2_ could reduce the stomatal density in 74% of the species investigated, including *Arabidopsis* [95], indicating that the atmospheric CO_2_ could greatly influence stomatal development. In *Arabidopsis*, mutants in which stomatal development response to high CO_2_ level is impaired are used to explore the molecular mechanism in the process. Mutations in two β-carbonic anhydrase genes, *CA1* and *CA4*, which act upstream of CO_2_-controlled stomatal movement [96], result in an increase of stomatal index under elevated CO_2_ conditions, indicating an essential role of *CA1* and *CA4* in repressing stomatal formation when CO_2_ level is high [33]. The *EPF2* transcripts are greatly upregulated in wild type in response to elevated CO_2_ level compared to *ca1 ca4*. A proteomic analysis of extracellular (apoplastic) proteins combined with a survey of CO_2_-inducible gene expression further identified the subtilisin-like serine protease CRSP. CRSP could specifically cleave the pro-peptide EPF2, but not EPF1 or Stomagen [33]. The biologically active EPF2 inhibits stomatal development at the initial stage [16, 17]. The repression of stomatal formation by high concentrations of CO_2_ may primarily be due to the EPF2-mediated negative regulation pathway (Fig. 2), as mutations in either *EPF2* or *CRSP* cause an increased stomatal index in response to elevated CO_2_ [33], probably by the excessive availability of Stomagen [25, 34].

Another mutant that shows deregulation of the CO_2_-controlled stomatal development response is *high carbon dioxide* (*hic*). The *hic* mutant does not show an obvious stomatal phenotype under normal conditions, but under elevated CO_2_ both stomatal index and stomatal density are increased [78]. *HIC* encodes a putative 3-keto acyl coenzyme A synthase involved in the synthesis of very-long-chain fatty acids [78]. Consequently, the *hic* mutant is defective in cell-wall wax biosynthesis [78, 97]. How *HIC* influences the stomatal pathway is still poorly understood, but other mutants with modified epicuticular wax also show compromised stomatal development [78, 98]. The cuticular wax layer serves as a barrier between the leaves and the environment. It is possible that alteration of leaf wax influences the permeability of some signal compounds, which could be EPF peptides, under elevated CO_2_ levels. Alternatively, modification of the wax layer could have an impact on light absorption or water perception, both of which have an influence on stomatal development [65, 77, 79–83]. Further investigation of the *hic* mutant would help in revealing the downstream signaling pathway.

### Water

Water condition, like other environmental factors, is known to affect stomatal development (Fig. 1b), but very little is known about the detailed mechanism. Low water potential can be generated by either less water or high osmotic pressure. It has been reported that water stress from soil (drought) reduces stomatal number in grasses [67, 79]. *Arabidopsis* under osmotic stress also exhibits reduced stomatal density [99]. High osmotic pressure destabilizes SPCH protein via the MAPK cascade and therefore results in fewer stomata on the *Arabidopsis* epidermis [99]. The negative regulation of water deficiency on stomatal development suggests an attractive idea of enhancing plant drought tolerance by manipulating stomatal density. Indeed, overexpression of EPF2 and EPF1 reduced stomatal density and improved drought tolerance in *Arabidopsis* and barley, respectively [100–102]. On the other hand, it is reported that moderate water deficits from soil have positive effects on stomatal number in grasses [79], indicating that plants precisely regulate water-use-efficiency via controlling stomatal development to optimize growth. Further investigations are required to understand how plants fine-tune stomatal formation in response to various water conditions.

## Unraveling signal integration

Stomatal development is influenced by multiple environmental and internal (hormonal) cues. Since the discoveries of the core stomatal signaling pathways and master regulators of stomatal differentiation [11], increasing efforts have been taken to delineate how these multiple inputs feed into the core stomatal pathways as we summarized in this review. Many questions remain open, however. For instance, environmental signals are perceived differently in specific tissue and cell types [103]. Expanding the cell state-specific profiling [104] under different environmental conditions may help decipher the molecular intersections in regional- and cell-state-specific contexts.

While key regulators of stomatal development are deeply conserved across land plants [9, 105], each plant species could exhibit a unique response to a given environmental condition, reflecting their natural history. Recent studies in grass stomatal development, for instance, revealed a neo-functionalization of stomatal bHLH proteins. For instance, a *MUTE* ortholog of *Brachypodium distachyon* acquired an additional function to initiate subsidiary cell division to form a stomatal complex unique to grass species [106, 107]. Unraveling how the integration of environmental and hormonal signaling pathways is rewired in different plant species to control stomatal development may shed light on their unique adaptive strategies. Using extremophytes, such as halophytes and aquatic heterophylly plant species, as a model may provide new insight.

It is important to emphasize that many of the molecular genetic studies of stomatal development were conducted in ‘idealized’ laboratory conditions. In nature, plants are constantly challenged by combinations of multiple stresses. While we did not cover it in this review, biotic stresses (pathogens) influence stomatal dynamics [108] and, possibly, stomatal development. There are at least two points of intersection between the pathogen signaling pathway and the core stomatal development pathway: the SERK receptors and the MPK3/6 kinases [14, 35, 109]. Future studies on signal integration will deepen our understanding of how plants optimize stomatal formation in ever-changing climates.
